# Liver function test indices-based prediction model for post-stroke depression: a multicenter, retrospective study

**DOI:** 10.1186/s12911-023-02241-0

**Published:** 2023-07-19

**Authors:** Jun Gong, Yalian Zhang, Xiaogang Zhong, Yi Zhang, Yanhua Chen, Huilai Wang

**Affiliations:** 1 Department of Information Center, University-town Hospital of Chongqing Medical University, Chongqing, China; 2 Medical Data Science Academy, Chongqing Medical University, Chongqing, China; 3 Department of Rehabilitation, Children’s Hospital of Chongqing Medical University, Chongqing, China; 4 National Clinical Research Center for Child Health and Disorders, Chongqing, China; 5 NHC Key Laboratory of Diagnosis and Treatment on Brain Functional Diseases, The First Affiliated Hospital of Chongqing Medical University, Chongqing, China; 6 College of Basic Medicine, Chongqing Medical University, Chongqing, China; 7 Department of Information Center, Rehabilitation Hospital of Chongqing Medical University, Chongqing, China; 8 Department of Pain and Rehabilitation, The Seventh People’s Hospital of Chongqing, Chongqing, China

**Keywords:** Post-stroke depression, Liver function test, Relationship, Predictors, Prediction model

## Abstract

**Background:**

Post-stroke depression (PSD) was one of the most prevalent and serious neuropsychiatric effects after stroke. Nevertheless, the association between liver function test indices and PSD remains elusive, and there is a lack of effective prediction tools. The purpose of this study was to explore the relationship between the liver function test indices and PSD, and construct a prediction model for PSD.

**Methods:**

All patients were selected from seven medical institutions of Chongqing Medical University from 2015 to 2021. Variables including demographic characteristics and liver function test indices were collected from the hospital electronic medical record system. Univariate analysis, least absolute shrinkage and selection operator (LASSO) and logistic regression analysis were used to screen the predictors. Subsequently, logistic regression, random forest (RF), extreme gradient boosting (XGBoost), gradient boosting decision tree (GBDT), categorical boosting (CatBoost) and support vector machine (SVM) were adopted to build the prediction model. Furthermore, a series of evaluation indicators such as area under curve (AUC), sensitivity, specificity, F1 were used to assess the performance of the prediction model.

**Results:**

A total of 464 PSD and 1621 stroke patients met the inclusion criteria. Six liver function test items, namely AST, ALT, TBA, TBil, TP, ALB/GLB, were closely associated with PSD, and included for the construction of the prediction model. In the test set, logistic regression model owns the AUC of 0.697. Compared with the other four machine learning models, the GBDT model has the best predictive performance (F1 = 0.498, AUC = 0.761) and was chosen to establish the prediction tool.

**Conclusions:**

The prediction model constructed using these six predictors with GBDT algorithm displayed a promising prediction ability, which could be used for the participating hospital units or individuals by mobile phone or computer.

**Supplementary Information:**

The online version contains supplementary material available at 10.1186/s12911-023-02241-0.

## Introduction

Stroke is a common clinical disease in the middle-aged and elderly. It is the brain tissue damage caused by a sudden rupture of a blood vessel in the brain or by a blockage in a blood vessel that prevents blood from flowing to the brain [1]. It is the leading cause of mortality and long-term disability among adults, and seriously threatening the health and lives of adults [2]. According to the Global Burden of Disease Study, stroke was the second leading cause of death in the world, and there are more than 2 million patients died because of stroke in China each year [3, 4]. In addition, a new survey showed that the absolute value of stroke events has increased by 70.0%, while the prevalence of stroke has increased by 85.0%. Moreover, the stroke-related deaths increased by 43.0% from 1990 to 2019 worldwide [5].

Previous studies showed the stroke patients not only have a significantly higher incidence of motor deficits, language problems, and intellectual disturbances than the general population, but also psychiatric complications [6]. Post-stroke depression (PSD), one of the most prevalent and serious neuropsychiatric effects of stroke, has been received widely attention. For example, a meta-analysis study involving 15,573 patients found that the prevalence of depression from 2 weeks to 7 years after stroke was as high as 33.5% [7, 8]. Another study showed there were more than half of stroke survivors experienced depression at some point after stroke [9]. Compared with the stroke patients, PSD patients was characterized with less participation in activities of daily living and social [10, 11], but more ideation and attempts of suicide [12, 13], cognitive impairment [14]. In addition, PSD can significantly up-regulated the risk of mortality and stroke recurrence after stroke [15, 16].

Presently, the diagnosis of PSD was relatively difficult, because some symptoms of PSD overlapped with stroke-related cognitive and functional changes [17]. Thus, many studies have explored the biomarker for PSD based on the peripheral blood, because it owes to the characteristics of convenient, economical, easy to obtain, and highly acceptable to patients. For example, a study found that serum thyrotropin of thyroid function could be a biomarker of PSD [18]. Another study found fibrinogen was positively correlated with the HAMD-17 score of PSD patients, while BDNF level was negatively correlated with the HAMD-17 score [19]. In addition, Zhao et al. found the elevated serum neurofilament light concentration was associated with higher risk of PSD [20]. Furthermore, Cheng et al. suggested the elevated serum levels of high-sensitivity C-reactive protein was associated with the risk of developing PSD, and PSD was closely related to immune inflammatory response [21]. Moreover, Li et al. proposed serum levels of homocysteine were an independent predictor of PSD [22]. However, these studies owed the features of limited sample size or the single center research.

Due to the incidence and severity of PSD, we need to find new risk factors to develop better preventive strategies. Liver, a major organ for substrate and energy metabolism, plays a crucial role in oxidative stress, glycogen storage, and synthesis of secreted proteins [23]. Previous studies showed it was closely associated with depression. For example, a study found the Chinese herbal medicines can effectively reduce depressive symptoms of PSD patients by regulating the liver function [20]. Other studies reported the liver disease was highly correlated with the depression and suicide attempts [24, 25]. Proteomic analysis revealed lipid networks were disturbed and the changes were specific in the liver of depression animal’s model [26–28]. Clinically, liver function test is a common blood test, and previous studies have found there was a correlation between the single liver function test indicators (such as AST, ALT, TBA, TBil, TP) and PSD or depression. While, there is little literature systematically assessing the relationship between the liver function test indices and PSD [29–32].

To address this issue, the purpose of this study was to assess the potential association between the liver function test indices and PSD by integrating the multi-center electronic medical data. To this end, a prediction model based on logistic regression and machine learning methods was also constructed. Furthermore, a series of indicators such as area under curve (AUC) were also performed to assess the performance of the prediction model.

## Materials and methods

### Study design

This study was a multi-center, retrospective study. The patients data were obtained from the health big data platform of Chongqing Medical University (Chongqing) of China (2015–2021), including the electronic medical records data of 7 hospitals of Chongqing Medical University. According to the international classification of diseases 10th edition (ICD-10), the stroke patients’ group (control group) included the discharge diagnosis of stroke, and the PSD patients’ group (study group) included the discharge diagnosis of depression based on the control group. The whole subjects were divided into the training set and test set by the ratio of 4:1 using random number table. The training set was used to select predictors and construct the prediction model, while the test set was used to verify the performance of the prediction model.

This study protocol was reviewed and approved by the Ethics Committee of the Chongqing Medical University with a waiver of informed consent. No potentially identifiable human images or data is presented in this study. All methods were performed in accordance with Declaration of Helsinki in 1964 and the relevant guidelines.

### Inclusion and exclusion criteria

The inclusion criteria were as follows: (1) age ≥ 18 years old; (2) ischemic or hemorrhagic stroke patients; (3) diagnosis was confirmed by computed tomography or magnetic resonance imaging. The exclusion criteria were as follows: (1) caused by brain injury, brain tumor or other non-vascular factors. (2) had pre-existing mental disorders, such as depression, schizophrenia; (3) missing all clinical information. PSD was diagnosed by a licensed psychiatrist using structured clinical interview and the Hamilton Depression Scale-17 score > 7.

### Data collection

The demographic characteristics and all admission first liver function test items were selected. Among them, the demographic characteristics include sex, age, smoke, drink. Liver function test indices included the γ-glutamyl transpeptidase (γ-GT), prealbumin (PAB), alanine aminotransferase (ALT), aspartate aminotransferase (AST), aspartate aminotransferase/alanine aminotransferase (AST/ALT), total bilirubin (TBil), total bile acid (TBA), total Protein (TP), albumin/globulin (ALB/GLB), globulin (GLB), albumin (ALB), direct Bilirubin (DBil), alkaline phosph-atase (ALP), indirect Bilirubin (IBil).

In the feature selection, T test was used to assess differences for normal continuous variables, and Mann­-Whitney U test was used to assess differences for non­-normal continuous variables and fisher exact test was used for categorical variables to perform univariate analysis. The least absolute shrinkage and selection operator (LASSO) and logistic regression analysis were performed for multi-variable analysis.

### Study population

**Fig. 1:**
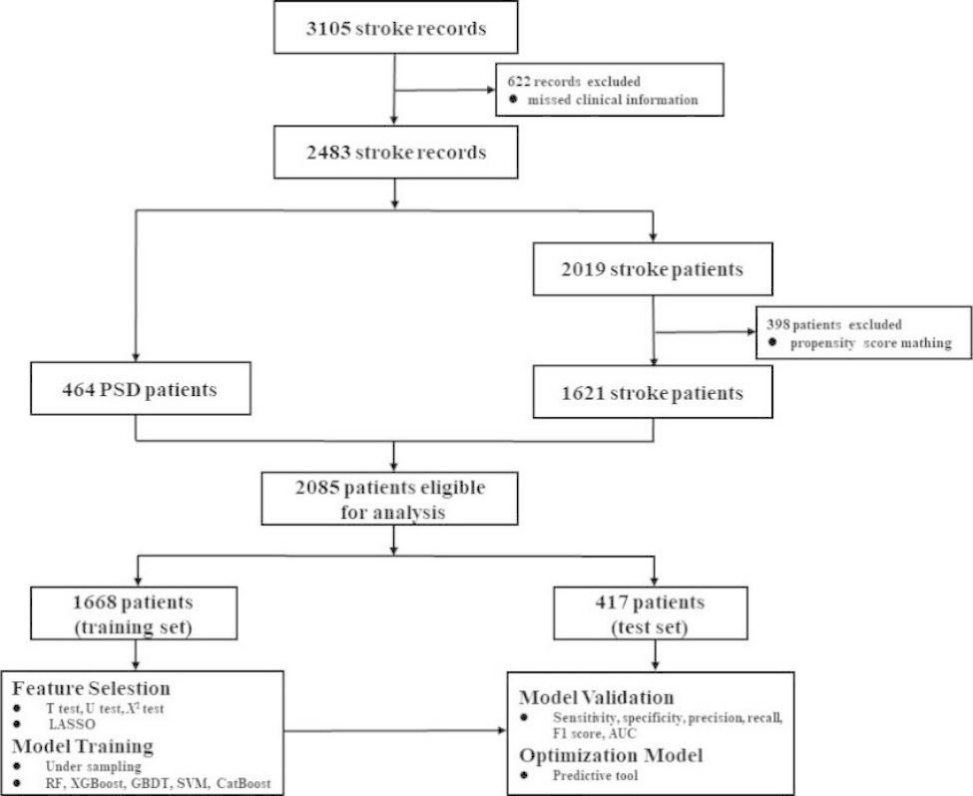
Flowchart of the patient’s selection *The abscissa is the magnitude of λ in LASSO regression model. The later the coefficient is compressed to zero as the lambda value varies, the greater the effect of the variable. Ten indicators were included when partial likelihood deviance was minimal*

A total of 3105 stroke patients’ electronic medical records were searched in the platform. Ultimately, 2085 patients met the inclusion criteria and were enrolled into the subsequent analysis, including 464 PSD patients and 1621 stroke patients. The training set included 1291 stroke patients and 377 PSD patients, while the test set included 330 stroke patients and 87 PSD patients. The specific screening process was shown in Fig. 1.

### Data analysis

All the statistical operations were carried out in R for Windows (version 4.0.3, https://www.r-project.org) and SPSS 25.0 (IBM Company of Armonk, New York, USA). Categorical variables were presented in counts and percentages, and continuous variables were presented in median and quartile ranges (IQR) or mean and standard deviations.

In the model construction, we firstly established a traditional logistic regression model based on the predictors after LASSO analysis. Furthermore, random forest (RF), extreme gradient boosting (XGBoost), categorical boosting (CatBoost), gradient boosting decision tree (GBDT), support vector machine (SVM) five machine learning algorithms were used to establish prediction models. In addition, we use the grid search algorithm to find and determine the optimal parameters of the machine learning algorithm. The grid search algorithm arranges and combines the possible parameters of the machine learning algorithm, puts each parameter combination into the model for calculation, and selects the optimal parameter from the parameter combination. The grid search results of the five model parameters can be found in Supplementary Table 1. The performance evaluation indicators including AUC, sensitivity, specificity precision, recall, F1 score. Calibration curve, decision curve and receiver operating characteristic curve were adopted to determine clinical practicability in the logistic regression model. Finally, we choose the model with the best performance to build a web-based prediction tool. *P < 0.05* was considered statistically significant in all statistical analyses.

## Results

### Differences in baseline

The quartile age for PSD patients and stroke patients was 66.0 (55.0, 74.0) and 66.0 (56.0, 75.0) respectively. For the PSD patients, 65.9% was male, 45.0% was smoker, and 41.4% was drinker. For the stroke patients, 63.1% was male, 40.8% was smoker, and 34.9% was drinker. There were no statistically significant variables between training set and test set. The details of variables were presented in the Table 1, and the target attribute of the variables used in this study were displayed in Supplementary Table 2.

**Table 1:** Baseline characteristics of the patients

Variables	PSD (N = 464) M (P25, P75)/N (%)	Stroke (N = 1621) M (P25, P75)/N (%)	Training set (N = 1668) M (P25, P75)/N (%)	Test set (N = 417) M (P25, P75)/N (%)	*p*-value
Age (year)	66.00 (55.00, 74.00)	66.00 (56.00, 75.00)	66.00 (55.00, 75.00)	66.00 (56.00, 74.00)	0.976
Gender (male)	306 (65.9)	1023 (63.1)	1081 (64.8)	256 (61.4)	0.213
Smoke (yes)	209 (45.0)	662 (40.8)	704 (42.2)	167 (40.0)	0.457
Drink (yes)	192 (41.4)	565 (34.9)	612 (36.7)	145 (34.8)	0.502
γ-GT (U/L)	33.06 (22.55, 50.25)	26.91 (18.00, 45.00)	28.69 (19.00, 47.70)	26.79 (18.84, 42.00)	0.144
PAB (mg/L)	229.03 (205.31, 260.01)	228.00 (188.05, 265.00)	228.94 (191.91, 264.60)	226.00 (192.99, 262.55)	0.686
AST (U/L)	20.45 (16.50, 25.99)	22.90 (18.44, 30.00)	22.14 (18.00, 29.00)	22.00 (18.00, 28.54)	0.744
ALT (U/L)	21.71 (16.07, 32.23)	19.00 (13.81, 28.00)	20.00 (14.00, 29.00)	19.00 (14.00, 28.01)	0.479
AST/ALT	0.90 (0.71, 1.17)	1.20 (0.95, 1.56)	1.14 (0.88, 1.46)	1.16 (0.90, 1.48)	0.527
TBA (µmol/L)	4.72 (3.81, 5.82)	3.55 (2.00, 5.45)	3.91 (2.20, 5.50)	4.13 (2.40, 5.91)	0.229
TBil (µmol/L)	10.22 (8.04, 13.14)	11.00 (8.20, 15.00)	10.90 (8.20, 14.53)	10.60 (8.10, 14.20)	0.514
TP (g/L)	67.60 (63.50,71.10)	69.08 (65.22, 73.78)	68.77 (65.00, 73.30)	68.30 (63.50, 72.70)	0.019
GLB (g/L)	27.12 (24.37 ,29.53)	28.90 (26.44, 31.40)	28.50 (26.04, 31.12)	28.20 (25.69, 30.97)	0.271
ALB (g/L)	40.48 (38.01, 43.01)	40.60 (37.44, 42.90)	40.62 (37.61, 43.00)	40.30 (37.30, 42.80)	0.095
ALB/GLB	1.49 (1.32, 1.69)	1.40 (1.27, 1.53)	1.41 (1.28, 1.56)	1.41 (1.27, 1.57)	0.636
DBil (µmol/L)	3.81 (2.80, 5.04)	4.10 (3.00, 5.70)	4.09 (2.95, 5.60)	3.85 (2.90, 5.70)	0.284
ALP (U/L)	79.21 (65.71, 94.77)	79.40 (66.00, 96.00)	80.50 (66.20, 97.00)	77.00 (65.00, 89.49)	0.004
IBil (µmol/L)	6.19 (4.70, 8.35)	6.80 (4.90, 9.50)	6.70 (4.89, 9.19)	6.60 (4.70, 9.00)	0.611

### Predictors selection

**Fig. 2:**
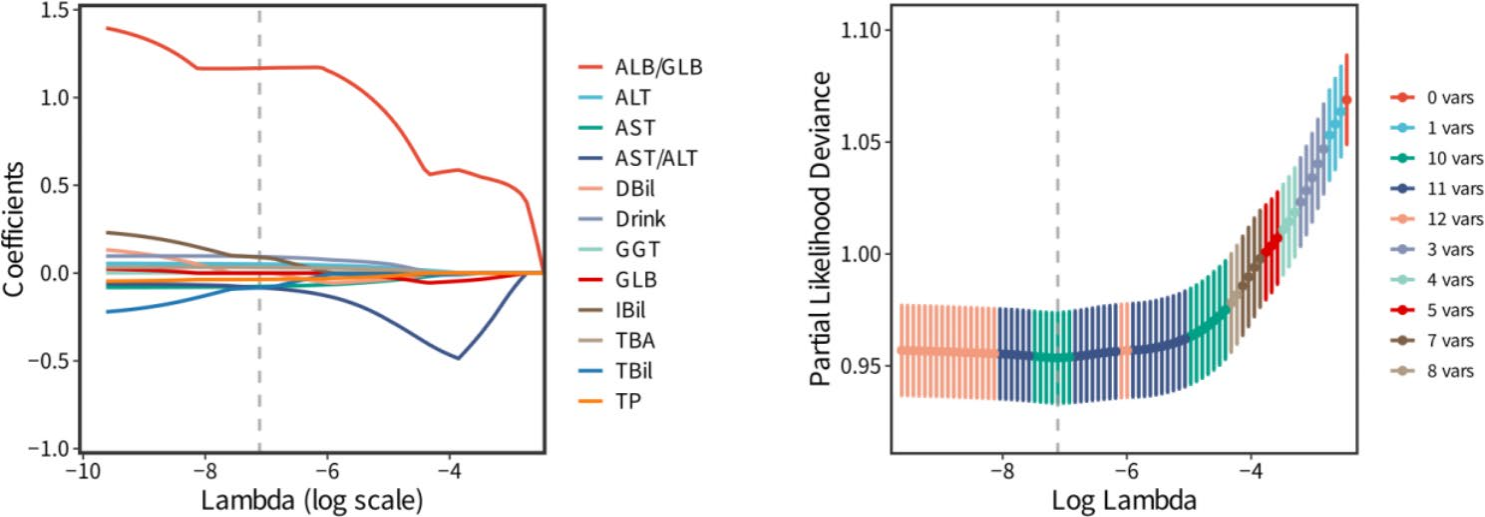
The LASSO analysis for potential predictors

Univariate analysis results showed there were twelve variables were statistically significant between the PSD patients and stroke patients in the training set (please see the Table 2). Subsequently, the indictors with statistical difference derived from univariate analysis were further selected by LASSO. The LASSO results suggested that the log of the optimal value of lambda was ten (please see the Fig. 2). Thus, ten variables were selected as the potential predictors for further analysis. They were drink, AST, ALT, AST/ALT, TBA, TBil, TP, GLB, ALB/GLB, DBil.

**Table 2:** General characteristics and univariate analysis from the training set

Variables	PSD patients (N = 377) M (P25, P75)/N (%)	Stroke patients (N = 1291) M (P25, P75)/N (%)	*P*-value
Age	66.00 (55.00, 74.00)	66.00 (55.00, 75.00)	0.281
Gender	247 (65.5)	834 (64.6)	0.790
Smoke (yes)	172 (45.6)	532 (41.2)	0.142
Drink (yes)	160 (42.4)	452 (35.0)	0.010
γ-GT (U/L)	31.94 (22.00, 48.70)	27.46 (18.00, 47.00)	< 0.001
PAB (mg/L)	229.80 (205.92, 259.87)	228.00 (188.00, 266.08)	0.213
AST (U/L)	20.28 (16.40, 25.99)	23.00 (18.58, 30.08)	< 0.001
ALT (U/L)	21.59 (15.80, 32.24)	19.47 (14.00, 28.00)	< 0.001
AST/ALT	0.89 (0.71, 1.17)	1.20 (0.95, 1.56)	< 0.001
TBA (µmol/L)	4.70 (3.73, 5.77)	3.50 (1.90, 5.40)	< 0.001
TBil (µmol/L)	10.26 (8.03, 13.00)	11.00 (8.25, 15.10)	0.001
TP (g/L)	67.90 (63.72, 71.40)	69.18 (65.59, 73.91)	< 0.001
GLB (g/L)	27.20 (24.39, 29.60)	28.90 (26.57, 31.40)	< 0.001
ALB (g/L)	40.64 (38.20, 43.16)	40.60 (37.50, 42.93)	0.153
ALB/GLB	1.50 (1.33, 1.69)	1.39 (1.27, 1.53)	< 0.001
DBil (µmol/L)	3.80 (2.78, 4.94)	4.20 (3.00, 5.70)	0.001
ALP (U/L)	79.23 (65.10, 95.09)	81.00 (67.00, 97.84)	0.339
IBil (µmol/L)	6.20 (4.71, 8.40)	6.90 (5.00, 9.41)	0.001

Subsequently, the potential predictors select by univariate analysis and LASSO analysis were used as the input variables, and whether PSD occurred in stroke patients was regarded as the target event (yes = 1, no = 0) to perform the binary logistic regression analysis (stepwise forward method). The results showed that six predictors, namely AST, ALT, TBA, TBil, TP, ALB/GLB were screened to enter the final model, please see the Table 3. The *β* values of ALT, TBA and ALB/GLB were 0.057, 0.029, 1.267, which were identified as risk factors. The β values of AST, TBil and TP were − 0.084, -0.027, -0.033, which were identified as protective factors. This proves that when the levels of ALT, TBA and ALB/GLB are increased, the probability of patients developing PSD will increase. On the contrary, when the levels of AST, TBil and TP are increased, the probability of patients developing PSD will correspondingly decrease.

**Table 3:** Logistic regression analysis for predictors

Predictors	*B*	*SE*	*P*-value	*OR (95%CI)*	*VIF*
AST	-0.084	0.009	< 0.001	0.920 (0.903–0.937)	3.056
ALT	0.057	0.006	< 0.001	1.059 (1.047–1.071)	2.834
TBA	0.029	0.008	< 0.001	1.030 (1.013–1.047)	1.127
TBil	-0.027	0.011	0.016	0.973 (0.951–0.995)	1.178
TP	-0.033	0.01	0.001	0.968 (0.949–0.987)	1.032
ALB/GLB	1.267	0.222	< 0.001	3.550 (2.296–5.490)	1.073

Furthermore, we conducted correlation analysis of the screened predictors. Results showed there was no correlation between TP and AST, ALT, TBA, while there exists a significant correlation among the left predictors, please see the Table 4. The variance inflation factor (VIF) of each predictor is less than 10 (Table 3), which suggests that there was no serious collinearity between the predictors.

**Table 4:** Correlation analysis of predictors

Predictors	AST	ALT	TBA	TBil	TP	ALB/GLB
**AST**	1	0.798^**^	0.195^**^	0.272^**^	0.034	-0.153^**^
**ALT**	0.798^**^	1	0.123^**^	0.157^**^	0.041	-0.047
**TBA**	0.195^**^	0.123^**^	1	0.298^**^	-0.004	-0.125^**^
**TBil**	0.272^**^	0.157^**^	0.298^**^	1	0.085^**^	-0.067^**^
**TP**	0.034	0.041	-0.004	0.085^**^	1	-0.145^**^
**ALB/GLB**	-0.153^**^	-0.047	-0.125^**^	-0.067^**^	-0.145^**^	1

*: The correlation is significant and *p* < 0.05; **: The correlation is significant and *p* < 0.01

### Model construction and validation

The six variables selected above were used to construct machine learning models. The test set was used to verify the ability of the established prediction model previously. In order to solve the problem of imbalanced data sets, we used random under-sampling to balance the data sets. 377 patients with PSD and 377 patients with stroke in training set were used to develop predictive model. Patients with PSD were used as the positive category in the machine learning algorithm and patients with stroke were used as the negative category in the machine learning algorithm.

**Fig. 3:**
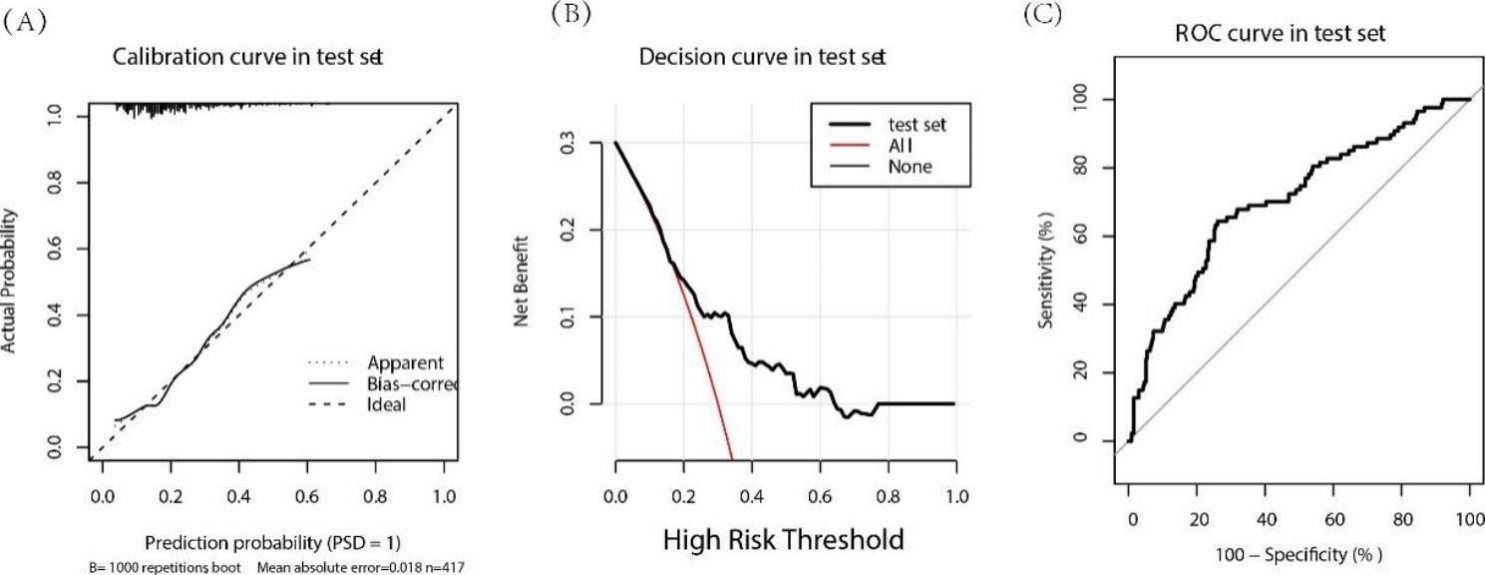
logistic regression model performance in the test set *The higher the SHAP value of the predictor, it proves that the metric has a greater contribution to the model*

In the logistic regression model, the performance of the prediction model showed the sensitivity, specificity was 0.598, 0.748 in the test set. ROC curve was adopted to assess the discriminatory capacity of the prediction model, and its AUC was 0.697 in the test set, which indicated a moderate performance. The calibration curve analysis plot was close to the ideal diagonal line, and decision curve analysis plot showed significantly better net benefit in the model both in the test set, which featured a moderate clinical practicability (please see the Fig. 3A C).

Table 5 shows the predictive performance parameters of five machine learning algorithms in the test set. Among the five models, CatBoost had the highest sensitivity (0.747), SVM had the highest specificity (0.688), GBDT had the highest F1 value (0.498). F1 is an indicator reflecting the positive prediction ability of the model, calculated by precision and recall. In our models, the highest F1 was 0.498, which proved that our model had ordinary performance in predicting PSD patients. This means that part of stroke patients was misdiagnosed as PSD in our model. In addition, AUC is the most comprehensive measure of model performance. The RF model achieved the highest AUC of 0.763 and higher than the GBDT’s 0.761. However, Delong test found that there was no significant difference in AUC between the RF model and GBDT model. In summary, it can be seen that the GBDT model outperforms the other machine learning and logistic regression models in the aspect of F1 and AUC, and there is no difference in AUC compared with RF model. Based on the comprehensive consideration of the prediction performance, we choose GBDT model to build a web-based prediction tool to predict the occurrence of PSD.

**Table 5:** Six algorithms’ model performance in the test set

Models	Sensitivity	Specificity	Precision	Recall	F1 score	AUC
XGBoost	0.678	0.682	0.360	0.678	0.470	0.720 (0.661–0.779)
RF	0.724	0.667	0.364	0.724	0.484	0.763 (0.710–0.815)
CatBoost	0.747	0.658	0.205	0.977	0.339	0.743 (0.689–0.797)
GBDT	0.736	0.679	0.376	0.736	0.498	0.761 (0.707–0.816)
SVM	0.667	0.688	0.360	0.667	0.468	0.677 (0.622–0.733)
Logistic regression	0.598	0.748	0.382	0.600	0.467	0.697(0.633–0.762)

**Fig. 4:**
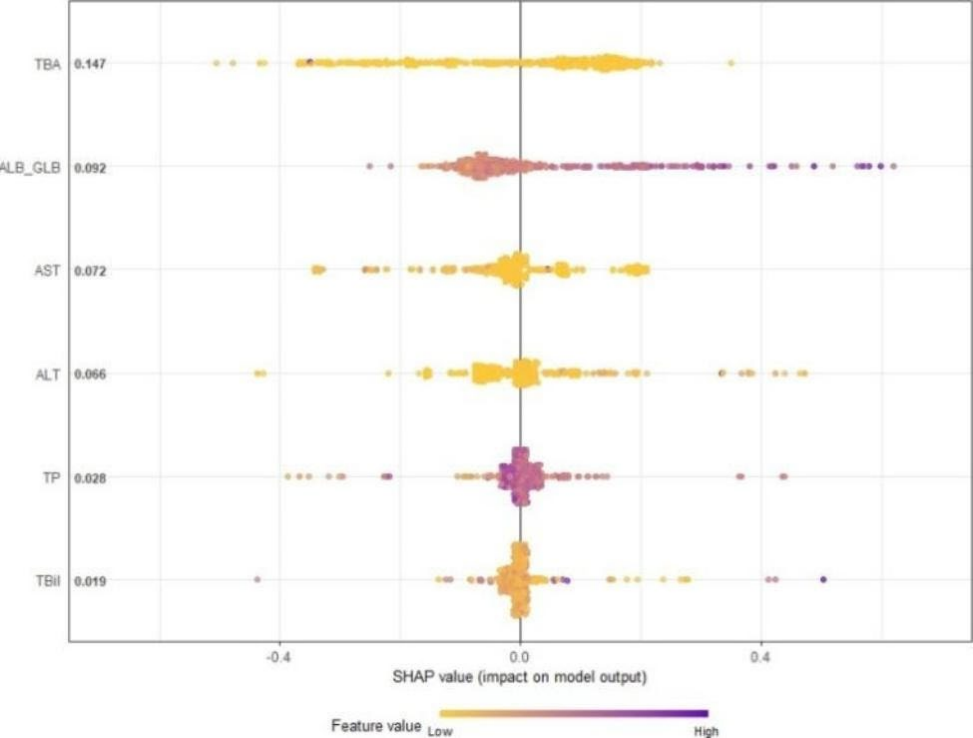
SHAP value based on predictors

Furthermore, we also calculated the SHapley Additive exPlanation (SHAP) values for each predictor, please see Fig. 4. The higher the SHAP value of the predictor, it proves that the metric has a greater contribution to the model. Therefore, the importance of the predictors to the model was ranked as follows: TBA, ALB/GLB, ALT, AST, TP, TBil, and the corresponding SHAP values were 0.151, 0.092, 0.067, 0.063, 0.030, 0.017.

## Discussion

In this study, we retrospectively collected 1668 stroke and 417 PSD electronic medical records from seven affiliated institution of Chongqing Medical University. Six indicators, namely AST, ALT, TBA, TBil, TP, ALB/GLB, were filtered based on the demographic characteristics and the liver function test indices. Finally, a prediction model was constructed for the diagnosis of PSD.

Here, we gathered a multi-center liver function test indices based on the electronic medical records, and we observed AST, ALT, TBA, TBil, TP, ALB/GLB could be the predictors of PSD. AST was mainly found in the mitochondria of liver cells, and it is released into the blood if liver cells are damaged. Alaine, aspartate and glutamate metabolism has been found closely associated with depression, as L-Aspartate could be catalyzed by AST to L-Gglutamine [33, 34]. Previous studies have found that animal models of depression have lower levels of aspartic acid and higher levels of L-glutamine in their plasma and serum [35]. As for ALT, it was widely existing in the cytoplasm of liver cells. Our results showed the ALT level was significantly elevated in PSD patients compared with the stroke patients. A study from the Korea showed the reduced ALT was an independent risk factor that increase the mortality in the elderly after ischemic stroke [29]. While, a prospective cohort from the UK biobank revealed the elevated ALT was closely associated with 5-year major depressive disorder (MDD) incidence [30]. As for the TBA, the previous studies showed the bile acid biosynthesis plays a crucial role in the development of depression [31, 32]. For example, a study from China showed the 23-nordeoxycholic acid in MDD patients was significantly higher than in healthy controls [36], and the antidepressant effects of fluoxetine appeared to be involved in bile acid metabolic pathway [37]. TBil is the sum of direct bilirubin and indirect bilirubin, which is an important index to reflect the jaundice severity. Previous studies showed the high bilirubin level is associated with the risk of PSD [38], which was contrary to the results of ours. The possible explanation was the author used a qualitative data mainly focused on bilirubin level more than 14.1 mmol/L. Furthermore, another study found the direct bilirubin was increased in MDD patients, while the indirect bilirubin was decreased in persistent PSD patients [39]. As for the TP, it has the functions of maintaining normal colloid osmotic pressure and PH of blood, transporting a variety of metabolites, regulating the physiological effects of transported substances and removing their toxicity, immunity and nutrition. An observational study from Sweden found low serum ALB was associated with depressive symptoms in elderly individuals’ long-term PSD [40]. Furthermore, a study including 307 acute stroke patients found the serum prealbumin level was associated with PSD after one month [41]. While, the ALB level was not statistically significant in our study.

Recently years, machine learning algorithms have been widely applied in the medical field, becoming a powerful tool for dealing with many healthcare problems. In this study, the GBDT-based PSD prediction model had the best performance (F1 = 0.498, AUC = 0.761), which indicated that our model had a promising performance in predicting PSD. At present, many scholars have studied the use of advanced methods to accurately identify PSD in stroke patients, please see Supplementary Table 3. Among the five studies, the best performing model was Simon Ladwig’s model [42], with an AUC of 0.84(0.78 to 0.90). Compared with other studies, the prediction model constructed in this study achieved a medium clinical predictive performance. While, the liver function test items were more convenient, economical and rapid compared with the expensive testing instruments and complicated testing methods. More importantly, the sample size in this study guaranteed the stability of the prediction model.

Based on these six predictors filtered in this study and GBDT model, we also developed a web-based tool (https://cqmugj.shinyapps.io/post_stroke_depression/), which could be used for the participating hospital units by mobile phone or computer. Once the stroke patients completed the liver function test, the clinicians or the caregiver can input these six indicators to obtain the probability of depression occurrence of stroke patients. For high-risk patients, reasonable intervention measures and treatment should be taken as soon as possible to avoid the deterioration of patients’ condition.

There are also some limitations in our study. Firstly, all of the data comes from the southwest of China, which may cause a selection bias. Secondly, we did not differentiate between ischemic stroke and hemorrhagic stroke patients in this study, so more subgroup analysis was needed in our follow-up work. Thirdly, the machine learning model developed in this study has moderate performance. It is necessary to screen for better predictors or adjust algorithms to improve model performance. Admittedly, many patients had complications (such as hypertension, diabetes et al.) other than stroke, while it was difficult to obtain these data due to the textual data. Therefore, more studies are needed to support our results.

## Conclusions

This study explored the correlation between the liver function test indices and PSD, identified several predictors of PSD, including AST, ALT, TBA, TBil, TP, ALB/GLB. Based on these predictors, a GBDT-based model and web-based tool for the early prediction of PSD was constructed, and the test set confirmed its clinical practicability.

## Electronic supplementary material

Below is the link to the electronic supplementary material.

Supplementary Material 1

Supplementary Material 2

Supplementary Material 3

## Data Availability

The data underlying this article will be shared on reasonable request to the corresponding author.

## Abbreviations

γ-GT: γ-glutamyl transpeptidase
PAB: Prealbumin
AST: Aspartate aminotransferase
ALT: Alanine aminotransferase
AST/ALT: Aspartate aminotransferase/alanine aminotransferase
TBA: Total bile acid
TBil: Total bilirubin
TP: Total Protein
GLB: Globulin
ALB: Albumin
ALB/GLB: Albumin/globulin
DBil: Direct Bilirubin
ALP: Alkaline phosph-atase
IBil: Indirect Bilirubin
